# Undiagnosed Systemic Lupus Erythematosus Presenting as Hemophagocytic Lymphohistiocytosis

**DOI:** 10.1155/2015/748713

**Published:** 2015-07-08

**Authors:** Ahmad K. Rahal, Justin Fernandez, Christopher Dakhil

**Affiliations:** ^1^Department of Internal Medicine, University of Kansas-Wichita, 1010 North Kansas Wichita, Wichita, KS 67214, USA; ^2^Cancer Center of Kansas, 818 North Emporia, No. 403, Wichita, KS 67214, USA

## Abstract

Hemophagocytic Lymphohistiocytosis (HLH) is rarely diagnosed in adults. Incidence is reported as one case per million persons per year. It can be triggered by conditions that affect immune homeostasis as infections, malignancies, and rheumatologic disorders. The following case demonstrates a rare instance in which undiagnosed systemic lupus erythematosus (SLE) presented as HLH. A 28-year-old male presented with progressive weakness and recurrent fevers for 2 months. Vital signs were within normal limits except for temperature of 100.3°F. His exam was unremarkable except for a left cervical scar and malar rash. His labs showed pancytopenia with neutropenia, hypertriglyceridemia, hypofibrinogenemia, and hyperferritinemia. Hemophagocytosis was present on bone marrow biopsy. All workup for a source of infection was negative. A tentative diagnosis of HLH was made based on clinical presentation and laboratory data. The patient was treated with an HLH protocol. Later, it was determined that his HLH was actually secondary to a primary diagnosis of SLE. The patient was treated for SLE with an immunosuppressive regimen of cyclosporine and dexamethasone, and he improved dramatically. HLH rarely presents due to a rheumatologic condition such as SLE. Physicians should consider testing for SLE in patients diagnosed with HLH.

## 1. Introduction

Hemophagocytic Lymphohistiocytosis (HLH) is an aggressive and life-threatening syndrome of excessive immune activation. Incidence is reported roughly as one case per million persons per year. It most frequently affects children and rarely adults [1]. HLH can occur as a familial primary form or secondary to a variety of events that disrupt immune homeostasis, such as infections, medications, neoplasms, and rheumatologic diseases [2]. HLH is characterized by the proliferation and activation of T lymphocytes and macrophages, which produce an excessive inflammatory response and hypersecretion of cytokines [1, 2]. Clinically, patients usually present with prolonged fever, pancytopenia, hepatosplenomegaly, liver function abnormalities, coagulopathy, and hyperferritinemia [2]. Systemic lupus erythematosus (SLE) is a systemic autoimmune disorder involving multiple visceral organs. In the adult population, HLH may be associated with SLE [3]. Prevalence of HLH secondary to SLE is estimated between 0.9% and 4.6% [4, 5]. We present a case that demonstrates a rare instance in which undiagnosed SLE presented as HLH.

## 2. Case Presentation

We report a 28-year-old Caucasian male who was in his usual state of excellent health until 2 months before presentation. He presented with progressive weakness, flu-like symptoms, and recurrent fevers. He had an enlarged and painless lymph node in the posterior part of his left neck. Core needle biopsy revealed that the lymph node was benign. Home medications included ibuprofen and acetaminophen as needed for fever. Review of systems was positive for hip pain bilaterally and negative for cough, diarrhea, weight loss, and night sweats. He denied any recent travel or exposure to animals. Vital signs were within normal limits except for temperature of 100.3°F. His exam was unremarkable except for a left cervical scar from the aforementioned lymph node biopsy and malar rash that started 4 days before presentation. His laboratory workup showed pancytopenia with neutropenia, abnormal liver function tests, hypertriglyceridemia, fibrinogen level of 126 mg/dL, and a ferritin level of more than 40,000 ng/mL. Bone marrow (BM) biopsy showed hemophagocytosis. Infectious workup was negative. A tentative diagnosis of HLH was made based on clinical presentation and the laboratory data, which satisfied the HLH-2008 diagnostic criteria [6]. The patient was transferred to a regional quaternary medical center and treated with an HLH protocol using etoposide and dexamethasone. Later, it was determined that his HLH was actually secondary to a primary diagnosis of SLE after a workup of his malar rash. The diagnosis of SLE was made with clinical/laboratory findings of malar rash, arthritis, pancytopenia, positive antinuclear antibody (ANA) test, positive antidouble stranded DNA (dsDNA) test, and low complement levels (C3, C4, and CH50). The patient fulfilled the American College of Rheumatology (ACR) and the Systemic Lupus International Collaborating Clinics (SLICC) classification criteria for SLE [7, 8]. The patient was treated for SLE with an immunosuppressive regimen of cyclosporine and dexamethasone, and he improved dramatically.

## 3. Discussion

The patient in our case presented with pancytopenia, neutropenia, and fever of 2-month duration. The differential diagnosis was broad including infections, malignancy, or autoimmune diseases. Infectious etiology was very high in the differential diagnosis and a complete infectious workup was done although negative. Blood and urine cultures were negative. C-reactive protein was less than 2.9 and erythrocyte sedimentation rate was 5. BM biopsy was sent for fungal cultures, acid fast bacilli stain and culture, gram stain, and viral culture but was negative. Serum was sent for human immunodeficiency virus, hepatitis panel,* Bartonella henselae*, Quintana IgG and IgM antibodies (ab), Blastomyces ab,* Brucella abortus* ab,* Coccidioides* ab,* Chlamydia burnetii* ab,* Cryptococcus* antigen (ag),* Cytomegalovirus* ab, Epstein Barr virus ab, Urine Histoplasma ag, Parvovirus ab, and* Toxoplasma* ab and all came back negative. Computerized tomography (CT) of the head/abdomen/pelvis and chest X-ray were negative for infection, masses, organomegaly but did show minimally prominent lymph nodes in the retroperitoneum and along the iliac chain; however, none were significantly enlarged by CT criteria. Once an infection etiology was ruled out, attention was turned to a workup of occult malignancy. As previously stated, imaging was negative, lactate dehydrogenase was elevated at 954 which suggested malignancy but peripheral smear did not show any blasts and peripheral blood flow cytometry was negative. BM biopsy was negative for carcinoma, lymphoma, leukemia, or granulomata but did show monocyte containing ingested red cells was consistent with hemophagocytosis. According to the HLH-2008 diagnostic criteria, the diagnosis of HLH is made by either molecular identification of an HLH-associated gene mutation or by fulfilling five of eight diagnostic criteria [6]. Our patient was diagnosed with HLH fulfilling five out of eight clinical/laboratory diagnostic criteria including fever, pancytopenia, hypofibrinogenemia, hemophagocytosis in BM, and hyperferritinemia. The diagnostic mainstay of HLH depends on the cytological findings of hemophagocytosis. Both BM biopsy and aspiration should be performed when HLH is suspected [9]. Our patient was treated for HLH and discharged home. Outpatient workup for his malar rash diagnosed him with SLE. The diagnosis of HLH secondary to SLE is complicated, because they have some features in common, but HLH is characterized by hyperferritinemia, hypofibrinogenemia, and hypertriglyceridemia, unlike SLE. Wong et al. [10] did a retrospective review of cases with unusual presentation of SLE and they found that 6 cases had HLH concomitant with SLE. Those 6 cases were not associated with an infectious etiology. Cytopenias are common manifestations of both HLH and SLE and the existence of HLH may lead to a delay to diagnose underlying SLE. Thus it is important to perform the immunologic testing for SLE in a setting of HLH and avoid diagnostic delay.

Treatment of primary HLH has become standardized based on the HLH-2004 protocol using cyclosporine, etoposide, and dexamethasone with or without intrathecal methotrexate followed by hematopoietic stem cell transplantation [2]. Treatment of secondary HLH is less well standardized but generally is aimed at treating the underlying condition. If unsuccessful, cytotoxic agents such as those in HLH-2004, steroids, intravenous *γ*-globulin, or targeted immune therapy have been used. In HLH due to SLE, corticosteroids and immunosuppressive agents have been used including cyclosporine, cyclophosphamide, intravenous immunoglobulin, and etoposide with variable success [11]. Keith et al. described the successful use of alemtuzumab to treat HLH due to systemic lupus erythematosus [12]. Infliximab was used successfully in a case of refractory HLH due to SLE [13].

## 4. Conclusion

Adult HLH usually presents secondary to infection but is rarely due to a rheumatologic condition such as SLE. Physicians should consider testing for SLE in patients diagnosed with HLH. This diagnosis should always be kept in mind when those with rheumatologic conditions acutely decompensate and present with multiple hematologic abnormalities.
